# Considerations for monitoring population trends of colonial waterbirds using the effective number of breeders and census estimates

**DOI:** 10.1002/ece3.4347

**Published:** 2018-07-20

**Authors:** Fagner M. da Silva, Carolina I. Miño, Rafael Izbicki, Silvia N. Del Lama

**Affiliations:** ^1^ Departamento de Genética e Evolução Universidade Federal de São Carlos São Carlos São Paulo Brazil; ^2^ Instituto de Biología Subtropical (IBS) Universidad Nacional de Misiones CONICET Posadas Misiones Argentina; ^3^ Departamento de Estatística Universidade Federal de São Carlos São Carlos São Paulo Brazil

**Keywords:** conservation genetics, effective size, microsatellites, *Mycteria americana*, simulations, single‐sample estimators

## Abstract

Detecting trends in population size fluctuations is a major focus in ecology, evolution, and conservation biology. Populations of colonial waterbirds have been monitored using demographic approaches to determine annual census size (*N*_a_). We propose the addition of genetic estimates of the effective number of breeders (*N*_b_) as indirect measures of the risk of loss of genetic diversity to improve the evaluation of demographics and increase the accuracy of trend estimates in breeding colonies. Here, we investigated which methods of the estimation of *N*_b_ are more precise under conditions of moderate genetic diversity, limited sample sizes and few microsatellite loci, as often occurs with natural populations. We used the wood stork as a model species and we offered a workflow that researchers can follow for monitoring bird breeding colonies. Our approach started with simulations using five estimators of *N*_b_ and the theoretical results were validated with empirical data collected from breeding colonies settled in the Brazilian Pantanal wetland. In parallel, we estimated census size using a corrected method based on counting active nests. Both in simulations and in natural populations, the approximate Bayesian computation (ABC) and sibship assignment (SA) methods yielded more precise estimates than the linkage disequilibrium, heterozygosity excess, and molecular coancestry methods. In particular, the ABC method performed best with few loci and small sample sizes, while the other estimators required larger sample sizes and at least 13 loci to not underestimate *N*_b_. Moreover, according to our *N*_b_/*N*_a_ estimates (values were often ≤0.1), the wood stork colonies evaluated could be facing the loss of genetic diversity. We demonstrate that the combination of genetic and census estimates is a useful approach for monitoring natural breeding bird populations. This methodology has been recommended for populations of rare species or with a known history of population decline to support conservation efforts.

## 1 INTRODUCTION

Assessing the size of natural populations is a major focus of population monitoring programs aimed at estimating population trends and determining priority species or areas for conservation (Lindenmayer & Likens, 2010). Changes in the census size of natural populations either over time (*N*_c_) or annually (*N*_a_) can indicate susceptibility to stochastic processes (Lande, 1988). However, the responsiveness of populations to evolutionary forces depends not only on population size, but also on genetic factors (Frankham, 1996). Therefore, measures of effective population size (*N*_e_) and effective number of breeders (*N*_b_) can be more informative than the census size itself (Trail, Brook, Frankham, & Bradshaw, 2010). *N*_e_ is the size of a Wright‐Fisher ideal population (Fisher, 1930; Wright, 1931) affected by genetic drift at the same rate as a real population (Crow & Kimura, 1970). *N*_b_ is a measure of *N*_e_ for a single breeding season, reflecting the parental contribution and changes in the inbreeding rate (Waples, 1990, 2002).

In natural populations, however, *N*_e_ cannot be estimated accurately most of the time, which is a major limitation to applying such approaches for population monitoring. The genetic and demographic parameters that affect *N*_e_ are often unknown in natural populations and most key assumptions of the available estimators may be violated (Wang, 2016). In populations of age‐structured species, estimates of *N*_b_ (${\hat{N}}_{b}$), which require data from a single cohort, are often more feasible to obtain than estimates of *N*_e_ (${\hat{N}}_{e}$) (Waples, 1990, 2002). ${\hat{N}}_{b}$ has been proposed to assess population trends and can provide crucial information for wildlife management and conservation, such as levels of genetic variation and the effects of ecological factors on population size (Hinkson & Richter, 2016; Schwartz, Luikart, & Waples, 2007; Whiteley et al., 2015).

Single‐sample methods using microsatellite information (for a review, see Luikart, Ryman, Tallmon, Schwartz, & Allendorf, 2010) have piqued the interest of researchers to estimate *N*_b_. However, these estimators are affected differently by population processes (e.g., immigration and genetic substructure) and methodological aspects (e.g., genotyping errors and sample size) (Belmar‐Lucero et al., 2012; Wang, 2016; Whiteley et al., 2012). Thus, evaluations of how these different factors influence ${\hat{N}}_{b}$ can determine the conditions under which this parameter is more reliable with regard to detecting changes in the size of natural populations (Fraser et al., 2007; Menéndez, Álvarez, Fernandez, Menéndez‐Arias, & Goyache, 2016). For example, the bias in ${\hat{N}}_{b}$ resulting from immigration depends on whether it is incidental or recurrent and on the genetic differentiation between focal and source populations (Fraser et al., 2007; Gomez‐Uchida, Palstra, Knight, & Ruzzante, 2013; Waples & England, 2011).

Many colonial waterbirds breed in sites where they depend on very specific environmental conditions and are subject to human‐induced changes that can result in variations in population size (Tsai, Reichert, Frederick, & Meyer, 2016; Vásquez‐Carrillo, Henry, Henkel, & Peery, 2013). Population sizes of colonial waterbirds have been monitored to evaluate changes in breeding colonies in space and time and are indicators of regional biodiversity, restoration success, and wetland quality (Atkinson et al., 2006; Kushlan, 1992; Péron, Ferrand, Leray, & Gimenez, 2013; Tavares, Guadagnin, Moura, Siciliano, & Merico, 2015). It would be ideal to count all birds in a colony at a given time, but census techniques most often do not enable the detection of real variations in the number of individuals. Colonial waterbirds, in particular, are difficult to monitor using conventional census because breeding colonies can be composed of thousands of breeding pairs, with large numbers of nonbreeding individuals, cryptic nests, and asynchronous reproduction. These problems can be minimized using genetic methods to estimate *N*_b_ from representative samples of the colonies. Likewise, monitoring approaches combining genetic and census estimates can provide insights into the role of life history on *N*_b_/*N*_a_ ratios and are recommended for the evaluation of the risk of stochasticity for population persistence (Belmar‐Lucero et al., 2012; Palstra & Fraser, 2012; Trail et al., 2010). However, prior to applying combined estimates in monitoring programs, an understanding of the relationship between ${\hat{N}}_{b}$ and ${\hat{N}}_{a}$ and the limits of such an approach is required (Bernos, Yates, & Fraser, 2018; Ferchaud et al., 2016). In this context, it is crucial to evaluate how genetic methods behave when estimating *N*_b_ under the conditions affecting natural waterbird colonies, which has not previously been investigated, and how to obtain robust census estimates in breeding colonies.

In this study, we used the wood stork, *Mycteria americana* (Linnaeus 1758; Ciconiiformes: Ciconiidae), as a model to evaluate the competence of estimates of *N*_a_ (${\hat{N}}_{a}$) and ${\hat{N}}_{b}$, and, indirectly, ${\hat{N}}_{b}/{\hat{N}}_{a}$ ratios for monitoring population trends. Characteristics of the wood stork make it a good model for these purposes: It is very sensitive to environmental changes in the wetlands where it inhabits and reproduces (Frederick, Gawlik, Ogden, Cook, & Lusk, 2009; Tsai et al., 2016); it exhibits colonial behaviour during the breeding season and is philopatric to its breeding sites, although to a lesser extent than are other species of Ciconiiformes (Frederick & Ogden, 1997); and it has a long prefledgling period (Bryan, Snodgrass, Robinette, & Hopkins, 2005), which enables sampling distinct generations. The fact that breeding colonies are often established in the same locations over time enables the possible assessment of population trends and indirect inferences regarding changes in such areas.

Our primary aim was to provide baseline methodological guidelines for monitoring changes in population size of colonial waterbirds by assuming breeding colonies as attractive target units for conservation, using both census and genetic methods. Using simulations, we analyzed different empirical datasets to illustrate the common problems encountered when estimating *N*_b_ in natural populations, particularly regarding sample size and number of loci. We assumed that a suitable genetic estimator of *N*_b_ for monitoring changes in population size of colonial waterbirds should (a) yield estimates proportional to ${\hat{N}}_{a}$, (b) have the power to yield precise estimates from datasets based on a limited number of genetic markers, and (c) require a sample size that can be collected during the short period of the breeding season at several sites. Furthermore, we apply our findings on the estimation of *N*_b_ to natural populations and we test the suitability of a counting method with an adjustment for the heterospecific composition of breeding colonies.

## 2 MATERIALS AND METHODS

### 2.1 Sample collection

**Figure 1:**
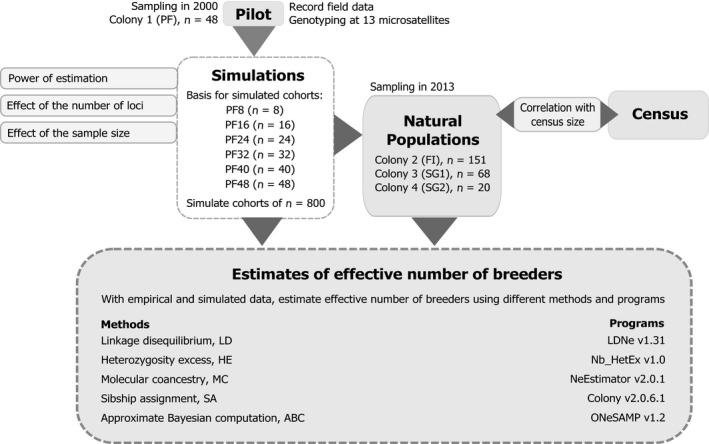
Flowchart outlining the methodological procedure followed. Original multilocus genotypes from Colony 1 and its subsets were used as basis to simulate large cohorts, in which predictions and effects of methodological issues in estimation of effective number of breeders (*N*_b_) were evaluated using different approaches. Estimates of *N*_b_ for natural populations (Colonies 2, 3, and 4) were obtained following guidelines determined using simulations, and effects of methodological issues were evaluated using census (*N*_a_)

Wood stork nestlings were randomly sampled at breeding colonies established in the Brazilian Pantanal wetland: colony Porto da Fazenda (PF, latitude ‐16.46873º, longitude ‐56.1258º, *n *=* *48) was sampled in 2000, colony Fazenda Ipiranga (FI, latitude −16.42736º, longitute −56.62183º, *n *=* *151), colony Sangradouro 1 (SG1, latitude −16.30992º, longitude ‐57.04925º, *n *=* *68), and colony Sangradouro 2 (SG2; latitude −16.31898º, longitude ‐57.04716º, *n *=* *20) were sampled in 2013 (Figure 1). The nests were accessed using ladders and climbing techniques. The nestlings were placed in bird bags, moved from the nests to a table on the ground where banding and blood collection took place, and returned safely to their respective nests. Blood (~0.2 ml) was collected from the brachial vein, using syringes rinsed with anticoagulant (EDTA 0.3%). The number of nestlings from each accessed nest containing at least one nestling (*k*) was recorded and adherence to Poisson's distribution (α < 0.05) was tested. The mean and the variance of the recorded values of *k* ($\overset{¯}{k}$ and *V*_*k*_, respectively) were calculated for each colony and used to determine the index of variability (*V*_*k*_ */* $\overset{¯}{k}$) (Crow & Morton, 1955).

### 2.2 Genetic analyses

Genomic DNA was extracted using the DNeasy^®^ Blood & Tissue kit (Qiagen). Samples were amplified at 13 species‐specific microsatellite loci (Supporting Information Table S1) and genotyped in a MegaBACE^®^1000 sequencer (GE Healthcare). Genotypes were recorded using Fragment Profiler ^®^ v1.2 (GE Healthcare), checked for evidence of null alleles using Micro‐Checker v2.2.3 (van Oosterhout, Hutchinson, Wills, & Shipley, 2004), and checked for identical multilocus genotypes using Colony v2.0.6.1 (Jones & Wang, 2010). Locus WS6 was reliably recorded only in PF and maintained in the simulation dataset, but had an average of 49.79% of missing data in other breeding colonies and was excluded from subsequent analyses. Identical multilocus genotypes were maintained in the datasets to test their effects on the estimation of *N*_b_ (Supporting Information Table S2).

### 2.3 Composition of datasets and common assumptions for estimation of *N*_b_

The informative content of each locus was estimated as 1—probability of identity using GenAlEx v6.5 (Peakall & Smouse, 2012). The mean number of alleles and mean expected heterozygosity was computed in Arlequin v3.5.2.2 (Excoffier & Lischer, 2010), and mean allelic richness was computed in hp‐rare v1.0 (Kalinowski, 2005); these indices were compared among all datasets. Linkage disequilibrium between loci and departures from Hardy‐Weinberg (HW) expectations were assessed in Arlequin v3.5.2.2 (Excoffier & Lischer, 2010), adjusting *p*‐values (α = 0.05) with the Holm‐Bonferroni correction (Holm, 1979).

Compliance with common assumptions of estimators was evaluated in the datasets. Selective neutrality was verified in Lositan v1.0 (Antao, Lopes, Lopes, Beja‐Pereira, & Luikart, 2008). Genetic differentiation within and among colonies was investigated by analysis of molecular variance (AMOVA) in Arlequin v3.5.2.2 (Excoffier & Lischer, 2010). Population subdivision was further investigated using the Bayesian clustering algorithm in Structure v2.3.4 (Pritchard, Stephens, & Donnelly, 2000). The parameters for these analyses were as follows: sampling location as prior, an admixture model with correlated allele frequencies, five runs per *K* (1 to 4), 2 × 10^5^ runs of MCMC and 10^4^ of burn‐in. The Delta *K* evaluation (Evanno, Regnaut, & Goudet, 2005) was applied in Structure Harvester v0.6.94 (Earl & vonHoldt, 2012). Migration patterns were inferred using a Bayesian method (Rannala & Mountain, 1997) implemented in GeneClass v2.0 (Piry et al., 2004) to compute the probabilities of multilocus genotypes in a given colony being descendants of immigrant (α  =  0.01, re‐sampling procedure with 10,000 simulated individuals).

### 2.4 Simulations to estimate *N*_b_ and design of natural population study

To evaluate the variation in ${\hat{N}}_{b}$ under different conditions and define the experimental design to properly sample colonies during the second sampling effort, cohorts were simulated using a method based on empirical data (Figure 1). The PF population was chosen as our base sample to perform the simulations because it is larger and more stable in number of individuals over the years than other studied wood stork populations (e.g., colonies FI, SG1 and SG2). Cohorts were simulated by resampling the genotypes from PF to compose five subdatasets with different allele frequencies; then, sets of 30 independent large cohorts (*n *=* *800) were simulated from each sub‐dataset in kingroup v2.08 (Konovalov, Manning, & Henshaw, 2004), setting a 1:1 adult sex ratio. To better reflect the situation found in colonial waterbird populations, the simulation model was based directly on allele frequencies of the PF genotypic dataset and simulated genotypes were created by randomly drawing alleles. Given a set of population frequencies, alleles are drawn using the internal number generator from the computer and matching the random number to the frequencies; for example, given alleles A, B and C with frequencies of 0.4, 0.5, and 0.1, respectively, the simulation model chooses a random number (R) from 0 to 1 and chooses an allele as follows: 0 ≤ *R* ≤ 0.4: choose A, 0.4 ≤ *R* ≤ 0.9: choose B, and 0.9 ≤ *R* ≤ 1: choose C (Dmitry A. Konovalov, personal communication). Therefore, the genotype frequencies in simulated cohorts are in concordance with HW equilibrium and with the provided allele frequencies (Dmitry A. Konovalov, personal communication). Frequencies of null alleles were adjusted using the Brookfield 1 method (Brookfield, 1996).

Further simulations were carried out to investigate how genetic estimators of *N*_b_ would behave under the properties of the genotypic datasets encountered in waterbird colonies (Figure 1). The simulations included a low number of loci, to account for the low presence of microsatellites in avian genomes (Ellegren, 2013; Primmer, Raudsepp, Chowdhary, Møller, & Ellegren, 1997), and low allele frequencies, to account for the intrinsically low levels of polymorphisms in microsatellite found in waterbirds (Campanini, Sanches, Hatanaka, & Del Lama, 2012; Nunes, Efe, Freitas, & Bugoni, 2017; Peters, Omland, & Johnson, 2007). The effect of a small increase in the number of loci was evaluated using sets of 7, 10, and 13 loci for each set of simulated cohorts: the 10‐locus dataset was achieved by removing three randomly selected loci (WSμ 03, WSμ 09 and WSμ 20); subsequently, another three randomly selected loci (WS1, WSμ 14 and WSμ 24) were removed to obtain the seven‐locus dataset. To investigate the influence of sample size on ${\hat{N}}_{b}$, random samples of 5% (*n *=* *40), 10% (*n *=* *80), 15% (*n *=* *120), 20% (*n *=* *160) and 50% (*n *=* *400) were taken from the simulated cohorts derived from the original PF dataset. *N*_b_ was estimated for all simulations using five single‐sample methods (Figure 1). The linkage disequilibrium (LD) method was employed in LDNe v1.31 (Hill, 1981; Waples, 2006; Waples & Do, 2008), assuming random mating and using minimum allele frequencies of 0.02, 0.05, and 0.10. The heterozygosity excess (HE) method was used in Nb_HetEx v1.0 (Pudovkin, Zaykin, & Hedgecock, 1996; Zhdanova & Pudovkin, 2008) with 10,000 bootstrap iterations. For the approximate Bayesian computation (ABC) method in ONeSAMP v1.2 (Tallmon, Koyuk, Luikart, & Beaumont, 2008), the prior range was set as 2‐100 to save computational time (changing the upper prior limit to 500 did not significantly change the resulting estimates). The molecular coancestry (MC) method was run in NeEstimator v2.0.1 (Do et al., 2014; Nomura, 2008). The sibship assignments (SA) method implemented in Colony v2.0.6.1 (Jones & Wang, 2010; Wang, 2009) was used assuming monogamy and a medium sibship prior of 2.0 for each parent.

### 2.5 Estimation of *N*_a_, *N*_b_, and *N*_b_/*N*_a_ from natural populations

An improved field technique based on counting the number of active nests and using $\overset{¯}{k}$ values was used to estimate *N*_a_ and cohort size (*N*_1_) of colonies FI, SG1, and SG2. At breeding colonies, nests were classified as “with content confirmed visually from the ground” (*N*_CG_) or “with content not confirmed visually from the ground” (*N*_NG_). *N*_CG_ were classified as active (i.e., those containing eggs, nestlings or occupied by adults) or inactive. Active nests were classified as belonging to wood storks (*N*_WD_) or other species. To estimate the number of nests with contents that could not be confirmed from the ground, but were indeed active wood stork nests, the status of nests was visually checked from tree tops in randomly selected sub‐areas of the colonies. The frequency of active wood stork nests (*F*_WD_) within *N*_NG_ was estimated using the total number of nests with contents that could not be confirmed from the ground (*N*_NG*SA*_) and the number of active wood stork nests confirmed only when checking from tree tops (*N*_WDSA_) in the sub‐areas (i.e., *F*_WD_ = *N*_WDSA_/*N*_NGSA_). Using this frequency, the total number of active nests per wood stork population (*TN*_WD_) was estimated as ${\hat{\mathrm{TN}}}_{\mathrm{WD}}=\;N_{\mathrm{WD}}+\left(N_{\mathrm{NG}}F_{\mathrm{WD}}\right)$. ${\hat{N}}_{a}$ was estimated as *2*(${\hat{\mathrm{TN}}}_{\mathrm{WD}}$) considering an independent pair of reproductive adults per active nest, and the number of nestlings in cohorts (${\hat{N}}_{1}$) was estimated as $\overset{¯}{k}$ (*TN*_WD_). Based on Sahai and Khurshid (1995), the 95% confidence interval (CI) for ${\hat{N}}_{a}$ was estimated as:

$$\begin{array}{cc} 95\;\%{\text{CI}}_{\left({\hat{N}}_{a}\right)}=\; & 2N_{\mathrm{WD}}+2N_{\mathrm{NG}}\left(2F_{\mathrm{WD}}\right)\pm 1.96\sqrt{\left(4N_{\mathrm{NG}}^{2}\right)\left(\frac{N_{\mathrm{NG}}-N_{\mathrm{NGSA}}}{N_{\mathrm{NG}}-1}\right)\left(F_{\mathrm{WD}}\right)\left(\frac{1-F_{\mathrm{WD}}}{N_{\mathrm{NGSA}}}\right)} \end{array}$$

and the 95% CI for ${\hat{N}}_{1}$ was estimated as $95\;\%{\text{CI}}_{\left({\hat{N}}_{1}\right)}=\left(95\%{\text{CI}}_{\left({\hat{N}}_{a}\right)}\overset{¯}{k}/\right)2$. *N*_b_ was estimated for cohorts sampled from the FI, SG1, and SG2 colonies as well as the pooled dataset of the three colonies using the genetic estimators described above in item 2.4, and ${\hat{N}}_{b}/{\hat{N}}_{a}$ ratios were calculated. To estimate *N*_b_ using the SA method, empirical $\overset{¯}{k}$ values were set as the medium sibship prior for each parent (J. Wang, personal communication).

### 2.6 Evaluation of *N*_b_ estimators

Although simulations under ideal conditions are more appropriate for assessing the accuracy of ${\hat{N}}_{b}$, when the objective is to provide genetic monitoring guidelines for natural populations, one should prioritize the use of a precise estimator under conditions similar to those encountered in nature (i.e., nonideal conditions). Estimates with infinite values could compromise the monitoring of population sizes, making it impossible to compare estimates in time and space. Therefore, the applicability of the single‐sample methods for monitoring changes in population size was initially evaluated based on the precision of the estimates measured by the mean percentage of outliers (for simulations), the mean percentage of finite ${\hat{N}}_{b}$ and the mean percentage of ${\hat{N}}_{b}$ with narrower 95% CIs.

The minimum sample size needed for each method was defined as the smallest sample size yielding ${\hat{N}}_{b}$ that differed significantly with an error rate ≤10% from those obtained by sampling all individuals from the simulated cohort. The average error rate of the estimates was determined by computing the percentage of the differences between harmonic means of ${\hat{N}}_{b}$ obtained using different sample sizes, and the entire set of simulated cohorts and the standard errors were computed using 10,000 bootstrap iterations. For real cohorts, correlations between ${\hat{N}}_{b}$ versus ${\hat{N}}_{a}$ and sample sizes (*n*) were assessed. The precision of ${\hat{N}}_{b}$ was equated to its log root mean squared error (*log RMSE*) for simulated cohorts and its variance (*V*) for real cohorts (Beebee, 2009). All statistical analyses were performed in R v3.3.1 (R Development Core Team, 2016).

## 3 RESULTS

### 3.1 Description of microsatellite loci data and population parameters

Null alleles were detected at locus WSμ 03 only in PF (frequency: 0.12) and locus WSμ 20 in all colonies (frequencies: 0.13 in PF, 0.05 in FI, 0.14 in SG1 and 0.20 in SG2). However, loci WSμ 03 and WSμ 20 had high informative content (from 0.55 to 0.80 for WSμ 03 and 0.73 to 0.85 for WSμ 20), and these were maintained in the datasets with their allelic frequencies adjusted. After correcting for the presence of null alleles, only loci WSμ 08 and WSμ 23 in the FI colony and locus WSμ 23 in the SG1 colony departed significantly from HW expectations. There was no evidence of significant linkage disequilibrium or deviations from selective neutrality at any loci. Genetic diversity indices (Table 1) differed moderately among the simulated cohorts and weakly among the real cohorts. For example, 55% of the 27 pairwise comparisons of *A*_O_ were significant, 26% of comparisons of *A*_R_ were significant and 41% of comparisons of *H*_E_ were significant. AMOVA indicated that most nuclear variation (99.09%) was contained within populations (*F*_ST_ = 0.009, *p *<* *0.001). Likewise, Bayesian clustering analyses revealed low Delta *K* values, with all individuals admixed at *K *≥* *2, suggesting gene flow and no significant population subdivisions (Supporting Information Appendix S1).

**Table 1:** Genetic diversity indices for simulated cohorts and wood stork populations from Pantanal wetland. Mean genetic diversity indices were showed for wood stork population of Porto da Fazenda (PF) and subsets of different numbers of individuals genotyped (PF8, PF16, PF24, PF32 and PF40) as well as for the natural populations from Fazenda Ipiranga (FI), Sangradouro 1 (SG1) and Sangradouro 2 (SG2). Indices obtained for different numbers of individuals genotyped (*n*): observed number of alleles (*A*_O_), allelic richness (*A*_R_), and expected heterozygosity (*H*_E_)

Simulated genotypes													
Dataset		*A*_O_				*A*_R_ a				*H*_E_			
	*n*	7 loci	10 loci	13 loci	13 loci^b^	7 loci	10 loci	13 loci	13 loci^b^	7 loci	10 loci	13 loci	13 loci^b^
PF8	8	2.14	2.20	2.54	2.31	2.14	2.20	2.54	2.31	0.41	0.44	0.49	0.45
PF16	16	2.57	2.50	2.77	2.54	2.29	2.30	2.57	2.35	0.40	0.41	0.46	0.42
PF24	24	2.57	2.60	2.85	2.62	2.21	2.28	2.55	2.34	0.40	0.40	0.45	0.42
PF32	32	2.57	2.90	3.15	2.92	2.16	2.33	2.62	2.41	0.41	0.43	0.47	0.44
PF40	40	2.86	3.10	3.31	3.08	2.20	2.35	2.62	2.42	0.43	0.43	0.47	0.44
PF	48	3.00	3.20	3.31	3.15	2.29	2.44	2.60	2.39	0.42	0.43	0.43	0.44

Natural populations				
Pop.^c^	*n*	*A*_O_	*A*_R_ a	*H*_E_
FI	151	3.92	2.43	0.40
SG1	68	3.25	2.45	0.43
SG2	20	2.50	2.40	0.41

^a^Estimates of *A*_R_ were obtained by rarefaction method in hp‐rare (Kalinowski, 2005), using smallest number of gene copies at single locus (*n *=* *16 for set of genotypes used for simulations and *n *=* *22 for natural populations). ^b^Without adjusting allele frequencies for presence of null alleles. ^c^Obtained for genotypic datasets at 12 microsatellite loci.

Evidence of low migration was found among wood stork colonies from the Pantanal wetland, as indicated by the assignment of three individuals sampled in FI and one individual sampled in SG1 as belonging to the SG2 colony. The mean number of nestlings from accessed nests ($\overset{¯}{k}$) was 2.29 in FI, 2.13 in SG1, 2.12 in SG2, and 2 in PF. The seasonal variance in the number of nestlings (*V*_*k*_) was 0.48 in FI, 0.35 in SG1, 0.24 in SG2, and 0.51 in PF. Nonrandom variance in breeding success was also found: mean *V*_*k*_ */* $\overset{¯}{k}$ ranged from 0.11 to 0.25 and *k* values did not adhere to a Poisson distribution model in any population (α < 0.05).

### 3.2 Estimates of *N*_b_ from simulated data

**Figure 2:**
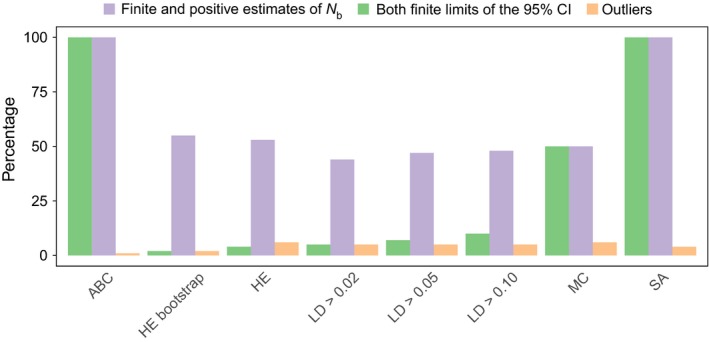
Power of different methods in estimating *N*_b_ for simulated cohorts. Mean percentage of all simulated cohorts that yielded finite and positive ${\hat{N}}_{b}$, finite 95% confidence interval (95% CI), and outliers are presented

**Figure 3:**
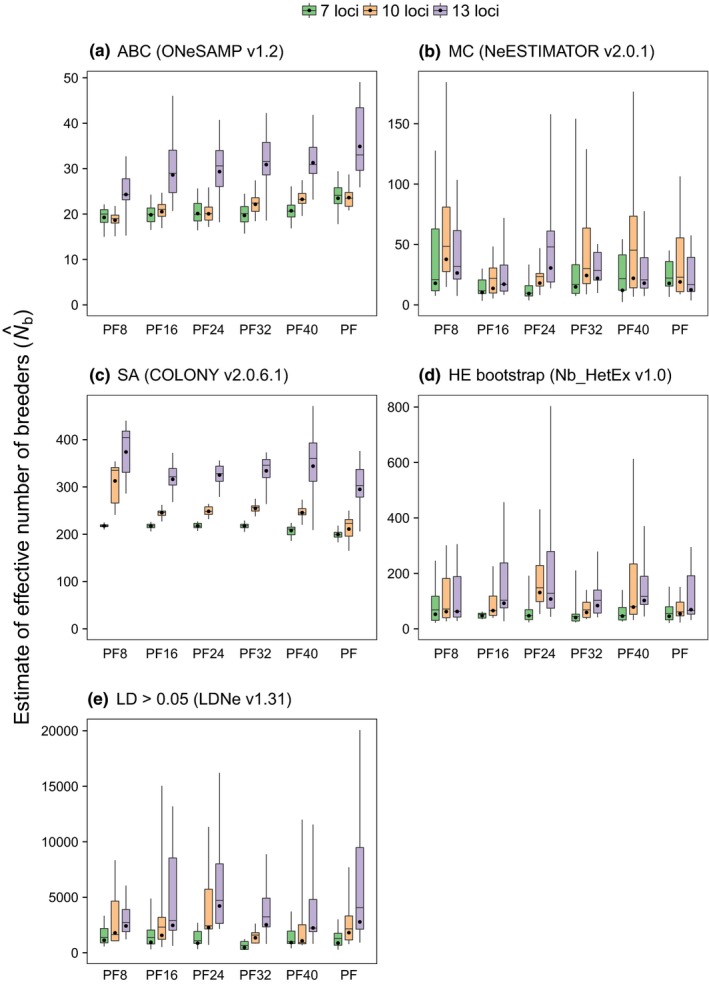
Estimates of effective number of breeders (${\hat{N}}_{b}$) for cohorts simulated from different datasets. ${\hat{N}}_{b}$ obtained for cohorts simulated for Porto da Fazenda (PF) population and subsets of different numbers of individuals genotyped (PF8, PF16, PF24, PF32, and PF40) are showed using following methods: approximate Bayesian computation (ABC; a), molecular coancestry (MC; b), sibship assignment with sibship size prior and sibship scaling (SA; c), heterozygote excess with 10,000 bootstrapping iterations (HE bootstrap; d), and unbiased linkage disequilibrium with allelic frequencies >0.05 (LD >0.05; e). Black dots within each bar indicate harmonic mean of ${\hat{N}}_{b}$

The ${\hat{N}}_{b}$ obtained for simulated cohorts revealed that the ABC and SA methods could be useful for estimating population sizes in waterbird colonies (Figures 2 and 3, Supporting Information Appendix S2). Only the ABC and SA methods yielded finite ${\hat{N}}_{b}$ with finite 95% CIs for all cohorts. There was no significant difference between the methods in the percentage of outliers (1 to 6%) (Figure 2). By yielding higher and more precise ${\hat{N}}_{b}$ when more loci were employed (Figure 3, Supporting Information Appendix S2), the ABC and SA methods also demonstrated a more consistent pattern of distribution of ${\hat{N}}_{b}$ obtained using different numbers of loci. Moreover, the ABC method, followed by the SA method, had the narrowest 95% CIs and *log RMSE* (Supporting Information Appendices S2 and S3). The HE and LD methods performed better with the re‐sampling approach and assuming a threshold of 0.05 for minimum allele frequencies, respectively. Only the results of these best approaches were considered in further evaluations with the HE and LD methods using simulations.

**Figure 4:**
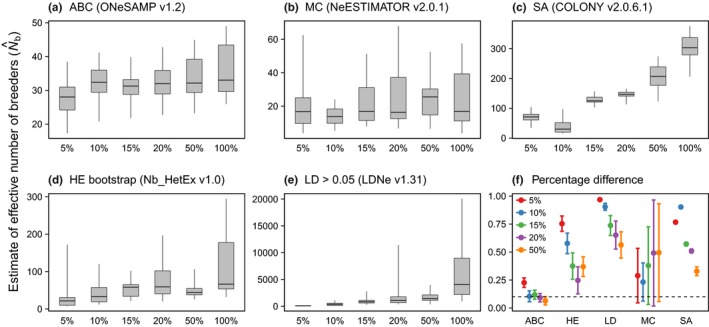
Estimates of effective number of breeders (${\hat{N}}_{b})$ obtained with different sample sizes. ${\hat{N}}_{b}$ values obtained by randomly sampling 5%, 10%, 15%, 20%, and 50% of entire cohorts simulated from original Porto da Fazenda (PF) population dataset. ${\hat{N}}_{b}$ values obtained by following methods are presented: approximate Bayesian computation (ABC; a), molecular coancestry (MC; b), sibship assignment with sibship size prior and sibship scaling (SA; c), heterozygote excess with 10,000 bootstrap iterations (HE bootstrap; d), and unbiased linkage disequilibrium with allelic frequencies >0.05 (LD >0.05; e). Percentage differences (error rate) calculated among harmonic mean of ${\hat{N}}_{b}$ obtained using different sample sizes versus harmonic mean of ${\hat{N}}_{b}$ obtained using entire cohorts (f). Dashed line indicates threshold of acceptable error rate (≤10%)

The minimum acceptable sample sizes determined for each estimator considering precision and error rates varied widely (Figure 4). The ABC method maintained an error rate of ≤10% with the smallest minimum sample size (≤10% of the population), whereas the LD method required a large sample size to maintain a low error rate. With a threshold of 10% for the error rate, the minimum sample sizes required by the SA and LD methods were >50% of the population. The minimum sample sizes required by the MC and HE methods were >10% and >20%, respectively. Comparisons of the ${\hat{N}}_{b}$ obtained by sampling 100% and 10% of the cohorts from the simulated populations revealed that the ABC method had the lowest error rate (10%), followed by the MC (23%), HE (58%), SA (90%), and LD (90%) methods (Figure 4).

### 3.3 Estimates of *N*_a_ and *N*_b_ for natural populations

The number of active nests, *N*_a_, and *N*_1_ are given in Table 2. ${\hat{N}}_{b}$ computed using different methods varied widely (Table 2). The ABC and SA methods yielded finite ${\hat{N}}_{b}$ values for all cohorts. The ABC method also yielded more precise estimates (narrower 95% CIs and lower *V*), followed by the SA, MC, LD, and HE methods (Table 2). ${\hat{N}}_{b}$ obtained using the ABC, SA and MC methods were positively correlated with ${\hat{N}}_{a}$ and sample sizes (correlation values = 1.0). ${\hat{N}}_{b}/{\hat{N}}_{a}$ ratios ranged from 0.02 to 0.49 (Table 2).

**Table 2:** Census data and estimates of effective number of breeders (${\hat{N}}_{b}$) in wood stork populations. Estimates and corresponding 95% confidence intervals (CI) for number of active nests, annual census of adults (${\hat{N}}_{a}$), and number of nestlings in cohorts (${\hat{N}}_{1}$) as well as ${\hat{N}}_{b}$, variance in ${\hat{N}}_{b}$ (*V*), and ${\hat{N}}_{b}/{\hat{N}}_{a}$ ratio were showed

Pop.	Active nests (95% CI)	${\hat{N}}_{a}$ (95% CI)	${\hat{N}}_{1}$ (95% CI)	Method	${\hat{N}}_{b}$ (95% CI)	*V*	${\hat{N}}_{b}/{\hat{N}}_{a}$
Fazenda Ipiranga (FI)	674 (637 – 710)	1,347 (1,274–1,420)	1,543 (1,459–1,626)	ABC^a^	30.60 (23.98–43.78)	64.71	0.02
				MC^b^	78.00 (0.1–391.7)	502.05	0.06
				SA^c^	78.00 (56–107)	65.38	0.06
				LD >0.02^d^	232.60 (110.9–1461.4)	580.61	0.17
				LD >0.05^e^	170.90 (87.6–591.6)	294.91	0.13
				LD >0.10^f^	149.50 (76.2–492.5)	278.46	0.11
Sangradouro 1 (SG1)	341 (327–354)	691 (654–708)	726 (696–754)	ABC^a^	24.70 (19.98–33.08)	53.04	0.04
				MC^b^	34.00 (0.9–125.6)	366.76	0.05
				SA^c^	52.00 (36–80)	84.62	0.08
				LD >0.02^d^	234.00 (68.6–∞)	NA	0.34
				LD >0.05^e^	332.10 (71.0–∞)	NA	0.49
				LD >0.10^f^	267.90 (60.9–∞)	NA	0.39
				HE^g^	15.00 (5.5–∞)	NA	0.02
				HE BTSP. h	13.00 (6.6–∞)	NA	0.02
Sangradouro 2 (SG2)	85	170	180	ABC^a^	18.60 (14.99–24.80)	52.74	0.11
				SA^c^	48.00 (26–124)	204.17	0.28
Pool of FI, SG1 and SG2	1,100	2,208	2,449	ABC^a^	31.64 (25.52–43.18)	55.81	0.01
				SA^c^	225.00 (181–277)	42.66	0.10

Notes

∞: infinite value; NA: not available.

^a^Approximate Bayesian computation (ONeSAMP v1.2). ^b^Molecular coancestry (NeEstimator v2.0.1). ^c^Sibship assignment without (Colony v2.0.6.1). ^d,e,f^Unbiased linkage disequilibrium with allele frequencies >0.02, >0.05, or >0.10, respectively (LDNe v1.31). ^g,h^Heterozygote excess with and without 10,000 bootstrap iterations, respectively (Nb_HetEx v1.0). ^i^Difference between finite limits of 95% CI as percentage of ${\hat{N}}_{b}$.

## 4 DISCUSSION AND CONCLUSIONS

### 4.1 Assumptions for estimation of *N*_b_

Genetic estimators of *N*_b_ make several simplifying assumptions, such as no immigration, no mutation, no selection, no population subdivision, and samples composed of discrete generations (Luikart et al., 2010; Wang, 2016). The paired comparisons using genetic diversity indices showed that the set of loci used provided sufficient discriminatory power to compute parameters associated with the estimation of *N*_b_ (Table 1). Likewise, most of the assumptions made by genetic estimators of *N*_b_ were met in the analyses of wood stork breeding colonies. For example, samples were collected at random from single cohorts representing nonoverlapping generations. Departures from a 1:1 adult sex ratio were not seen and assortative mating has not been reported in the wood storks (Coulter, Rodgers, Ogden, & Depkin, 1999). Given the low mutation rate of microsatellites in short time intervals (Beaumont, 2003), the observed departures from HW expectations in FI are unlikely to result from mutation and no evidence of selection was found (non‐significant neutrality tests). Furthermore, there was no evidence of significant population subdivision, as indicated by AMOVA and Bayesian clustering analyses (Supporting Information Appendix S1).

Evidence of migration was found among the studied colonies, albeit at a low rate, which is consistent with previous studies reporting regional philopatry with migration (Del Lama, Avelar, & Nascimento, 2015; Miño et al., 2017). However, if migrants are exchanged between genetically similar sources, the effects on ${\hat{N}}_{b}$ are expected to be small (Waples & England, 2011). Thus, the effect of migration on the estimation of *N*_b_ can be considered negligible in the colonies studied.

The observed variance in breeding success deviated significantly from the *V*_*k*_ */* $\overset{¯}{k}$ ≈ 1 ratio assumed by the Wright‐Fisher model (Waples, 2006). This is somewhat expected in the wood stork, because nonrandom variance in breeding success may be associated with failure to recruit individuals during the breeding season or the presence of age categories among breeders (Borkhataria, Frederick, Hylton, Bryan, & Rodgers, 2008). This variance in breeding success could reduce *N*_b_ in relation to the census size (e.g., Whiteley et al., 2015).

### 4.2 Estimates of *N*_b_ from simulated data

As reported for some methods in earlier studies (Pudovkin, Zhadanova, & Hedgecock, 2010; Wang, 2016; Waples & Do, 2010), the simulations made in this study suggest that single‐sample methods perform differently depending on the sample size and number of loci employed (Figures 3 and 4; Supporting Information Appendices S2 and S3). Under the conditions used herein, the ABC method demonstrated greater precision in estimating *N*_b_ (Figures 2 and 3; Supporting Information Appendices S2 and S3), which may be associated with the amount of information assessed using this method and the adequate choice of priors (Holleley et al., 2014; Luikart et al., 2010). Although the exact contribution of each summary genetic statistics used by the ABC method is unclear (Wang, 2016), ${\hat{N}}_{b}$ should be mainly influenced by the number of breeders producing cohorts (David A. Tallmon, personal communication). In contrast, the LD method demonstrated the most infinite values and lowest precision, possibly due to large sampling errors or very large *N*_b_ (Waples & Do, 2010). The high percentage of infinite estimates obtained by the HE and MC methods is consistent with previous studies showing that these methods most often yield finite values when the real *N*_b_ is very small (Pudovkin et al., 2010; Wang, 2016). Moreover, the ABC and SA methods demonstrated reduced *log RMSE*, hence increased precision, as more loci were used (Supporting Information Appendix S2). Few loci could lead to an increased effect of noise on the signal of genetic drift (Waples, 2006). The simulations made in this study are in line with this expectation, as few loci with low or moderate informative content originated some identical‐by‐state multilocus genotypes (Supporting Information Table S2) and resulted in lower ${\hat{N}}_{b}$ (Figure 3).

The ABC method seems to be less influenced by change in sample size, as indicated by its low error rate even when sampling only 10% of the population and the low rate of change in mean ${\hat{N}}_{b}$ with the increase in sample size (Figure 4). The SA method demonstrated more precise ${\hat{N}}_{b}$ (narrower 95% CIs and low error rate) when the sample size increased (Figure 4), as expected for a method that requires larger sample sizes when using markers with low informative content (Wang, 2009). The LD method also demonstrated greater precision with the increase in sample size (Figure 4). This is in agreement with the prediction that doubling the sample size improves the precision of this method more than doubling the number of loci (Antao, Pérez‐Figueroa, & Luikart, 2011). Larger sample sizes increase the number of alleles per locus used in the analyses and lower the odds of over‐sampling families (England, Luikart, & Waples, 2010; Whiteley et al., 2012).

### 4.3 Estimates of *N*_a_ and *N*_b_ for natural populations

Estimates of the effective number of breeders can provide important information on a population and complement census measures in monitoring programs (Ferchaud et al., 2016). The method used in the present study to estimate *N*_a_ in the wood stork proved to be promising for the population monitoring of colonial waterbirds, as it enabled determining the size of breeding colonies previously assumed to have different sizes by field observations alone. Moreover, ${\hat{N}}_{a}$ were useful for determining how many individuals corresponded to 10% of the sampled cohorts.

When the objective is to determine population trends using ${\hat{N}}_{b}$, it is desirable to have precise (even downward‐biased) estimates that are positively correlated with *N*_a_, but not with sample size (Beebee, 2009; Tallmon et al., 2010). In the present study, the ABC method yielded the most precise ${\hat{N}}_{b}$, followed by the SA method (Table 2), which is in line with previous studies on other species (e.g., Álvarez, Lourenço, Oro, & Velo‐Antón, 2015). As presented herein, Beebee (2009) also found greater variance (*V*) in ${\hat{N}}_{b}$ obtained with the SA method than with the ABC method, probably because setting prior limits to ${\hat{N}}_{b}$ in the ABC method leads to an increase in precision. On the other hand, the LD, MC, and HE methods demonstrated lower precision and none of these methods yielded finite estimates for SG1 or the pooled dataset of the three cohorts (Table 2). The ABC, SA, and MC methods fulfilled the criterion of positively correlating with ${\hat{N}}_{a}$, but also positively correlated with sample size. Using the SA method and setting $\overset{¯}{k}$ as the sibship prior improved ${\hat{N}}_{b}$ the with regard to reflecting the population size. Without the prior, however, larger populations yielded smaller estimates (data not shown). Given that natural populations with intrinsic moderate microsatellite variation were studied (Table 1), the results of the SA method could indicate that ${\hat{N}}_{b}$ may correlate with population size when loci information is insufficient, but $\overset{¯}{k}$ is known. The correlations with sample size found in the ABC method could probably arise because the sample size was set proportionally to ${\hat{N}}_{a}$, given that ${\hat{N}}_{b}$ obtained with this method were the only ones not to increase proportionally to sample size in the simulated cohorts (Item 4.2).

Although ${\hat{N}}_{b}/{\hat{N}}_{a}$ ratios varied widely, even in single colonies (Table 2), estimating these ratios in waterbird colonies may assist in long‐time monitoring to investigate demographic changes that may mirror concurrent environmental changes. The ${\hat{N}}_{b}/{\hat{N}}_{a}$ ratios (Table 2) were below the mean of 0.225 reported in a previous quantitative survey (Palstra & Fraser, 2012), except those calculated using ${\hat{N}}_{b}$ computed with the SA method for SG2 and the LD method overall. Nonetheless, ${\hat{N}}_{b}/{\hat{N}}_{a}$ ratios obtained in wood storks were often close to ${\hat{N}}_{e}/{\hat{N}}_{c}$ ratios reported for rare and endangered bird species (often <0.1) (e.g., Brekke, Bennett, Santure, & Ewen, 2011; Lopes, Miño, Rocha, Oliveira, & Del Lama, 2013; Ramstad, Colbourne, Robertson, Allendorf, & Daugherty, 2013).

### 4.4 Guidelines for monitoring populations of colonial waterbirds

Many species of colonial waterbirds nest in accessible aggregations, which is a condition that at first seems favorable to the application of census methods based on counts, as used in the present study, for monitoring population sizes (e.g., Brown, Tims, Erwin, & Richmond, 2001; Rush et al., 2015; Torres et al., 2010). However, census estimates can be difficult to obtain accurately when thousands of individuals are recruited during the breeding season or when breeding colonies occupy large areas that are difficult to cover in a short period of time. We recommend some characteristics to evaluate prior to choosing a counting method and a target for counting (e.g., active nests or breeding adults): (a) whether there are non‐breeding adults in the colony, (b) how easily nests and individuals can be observed for counting, (c) whether reproduction is asynchronous, which may require multiple visits to the colonies and the marking of individuals, (d) whether inactive nests that were abandoned or had persisted from previous breeding seasons may be confused with active nests by the presence of adults in the vicinity, and (e) the difficulty in observing the contents of nests from the ground. The use of genetic estimates, such as ${\hat{N}}_{b}$, is a valuable alternative for monitoring population size when there is insufficient information on the ecological traits and dynamics of breeding colonies, obtaining this information in the field would consume a prohibitively large amount of time, and when available information indicates that a census is not feasible.

Based on the workflow described in item 2 of this study, the monitoring using ${\hat{N}}_{a}$ and/or ${\hat{N}}_{b}$ can be divided into three consecutive steps. Step 1 (prior to field research) involves performing an extensive literature review, setting up a pilot study, and planning the field and laboratory research. Steps 2 and 3 (field and laboratory research) involve the execution of the planning to obtain the estimates of the census and effective number of breeders, following what was determined in the pilot study (i.e., applying the method(s) chosen for estimating *N*_b_ considering the available information on a given sample size and number of loci).

A literature review and an empirical enquiry near the location of the breeding colonies can eliminate some time‐consuming steps from the field research itself, which can assist in the planning of the monitoring as a whole. Using the wood stork populations sampled in the present study as an example, personal communications on the onset of the establishment of the colonies were crucial to defining the period in which the breeding colonies would be visited, as the more adequate time for collecting blood from wood stork nestlings is the period between three and six weeks of age. All information on the reproductive biology, genetic population structure, population dynamics, and impact of the human presence in the colonies (e.g., Bouton, Frederick, Rocha, Dos Santos, & Bouton, 2005; Del Lama et al., 2015; Frederick & Ogden, 1997; Miño et al., 2017) can contribute to answering important questions during field research planning, such as: 1—Which region and which colonies should primarily be sampled? 2—How much time can be spent visiting each colony? 3—What data can be recorded in the field? 4—What is the best way to count and record active nests considering the heterospecific composition of the colonies?

The results obtained in this study highlight the importance of conducting a pilot study to evaluate the quality of the genotypic dataset for the characterization of polymorphisms in loci, determine the smallest sample size needed to obtain a representative sample of the breeding colonies, and estimate the power of the marker set for confirming whether common assumptions for the estimation of *N*_b_ are fulfilled. A pilot study may start from either published data on populations of the species within the area of interest for monitoring, or from samples already collected and stored, as performed here using the PF sample and described in items 2.2, 2.3, and 2.4. In the absence of previous data or material, researchers can develop a pilot sampling expedition covering at least one colony in the focal region, which is a reasonable decision when considering long‐term monitoring.

Detailed planning for the monitoring of breeding colonies can optimize the use of funds and time, especially during the field work, when time can be a limiting factor to visiting several colonies in a single breeding season. Another aspect to consider during the field work is the strengthening of field efforts to minimize human disturbance within colonies and maximizing the information obtained when accessing nests (e.g., including banding, measuring individuals, collecting blood, recording demographic data to estimate reproductive success and census). Depending on the degree of natal philopatry of individuals to either colonies or regions, it is possible to monitor specific colonies or a geographic region. If birds exhibit high fidelity to the breeding sites, a researcher can monitor focal colonies and, in this case, the target should be larger colonies, which are most likely to persist over time than smaller ones (Tsai et al., 2016). Colonies that are not established in consecutive breeding seasons can be included when the monitoring covers a larger geographic range and the birds are philopatric to the region rather than to the colonies, as occurs with the wood stork (Miño et al., 2017). Moreover, breeding colonies should not be considered separate populations without testing for significant genetic differentiation or migration from dissimilar population sources prior to monitoring population size using ${\hat{N}}_{b}$. Similarly, to pool genetic samples confidently from different colonies as if they belong to a single panmictic unit, prior testing is necessary, as performed in the present study. The effects of ignoring underlining genetic structuring could be more harmful than those of estimating *N*_b_ separately for each colony, even if colonies are not structured (Bernos et al., 2018; Holleley et al., 2014).

The present study pointed that the ABC method is suitable for genetically tracking demographic changes in wood stork populations, as it required smaller sample sizes and fewer loci to estimate *N*_b_ with greater precision. Results also showed that the SA method is promising for the estimation of *N*_b_ when a sibship size prior can be set with certain confidence, for example, using the mean number of nestlings from accessed nests ($\overset{¯}{k}$). However, as demonstrated here, the SA method requires a larger sample size to yield precise estimates with a limiting number of loci. Likewise, findings from this study show that the sample size relative to *N*_a_ is an important and limiting factor to estimating *N*_b_. Therefore, whenever possible, researchers may estimate *N*_a_ and $\overset{¯}{k}$, in order to reach adequate sample sizes to obtain precise, unbiased ${\hat{N}}_{b}$. *N*_a_ and $\overset{¯}{k}$ should be estimated immediately before or concurrently with the blood collection during initial field work, as the number of nestlings in a colony may vary greatly over the breeding season (Borkhataria et al., 2008; Vergara & Aguirre, 2006).

This study demonstrates that *N*_a_ and/or *N*_b_ estimates are useful tools for monitoring natural breeding bird populations. However, as the estimates varied widely depending on the method used, researchers can follow the methodological workflow offered herein to explore genotypic datasets and choose the estimator most suited to the conditions of the focal natural populations prior to start the monitoring. Furthermore, this workflow may not provide useful for the effective monitoring of natural populations when it is not feasible in terms of funds and time to collect representative population samples or when monitoring populations of nomadic species. The methodology based on ${\hat{N}}_{a}$ described herein can be used by researchers interested in genetic monitoring and studying populations of birds with colonial breeding where the sampling of discrete generations is possible. In particular, the workflow offered herein can be used by researchers studying colonial birds with a limited number of microsatellites and low levels of polymorphism. Combining demographic and genetic estimates is a feasible strategy for monitoring few small colonies with no major access difficulties and low human impact. Moreover, combining demographic and genetic approaches is highly recommended for populations of rare species or with a known history of population decline to support conservation efforts by enabling the informed, timely, precise monitoring of trends in population size.

## CONFLICT OF INTEREST

None declared.

## AUTHOR CONTRIBUTIONS

F.M.S., C.I.M., and S.N.D.L. designed the study and wrote the manuscript. F.M.S. and S.N.D.L. collected samples. F.M.S. conducted the genetic analyses and the census in natural populations. F.M.S. and C.I.M. led the computational work. F.M.S. and R.I. conducted the statistical analyses and created the figures with the results.
