# Supplementary material for: Considerations for monitoring population trends of colonial waterbirds using the effective number of breeders and census estimates
Source: Ecol Evol. 2018 Jul 20;8(16):8088–101. doi: 10.1002/ece3.4347 (PMC6144984; doi:10.1002/ece3.4347)
Supplement: Supplementary file 1 [file ECE3-8-8088-s001.docx]

**SUPPORTING INFORMATION FOR ONLINE PUBLICATION**

**Considerations for monitoring population trends of colonial waterbirds using the effective number of breeders and census estimates**

Fagner M. da Silva^1*^, Carolina I. Miño^2^, Rafael Izbicki^3^, Silvia N. Del Lama^1^

^1^ Departamento de Genética e Evolução, Universidade Federal de São Carlos, CEP 13565-905, São Carlos, São Paulo, Brazil.

^2^ Instituto de Biología Subtropical (IBS), Universidad Nacional de Misiones, CONICET, N3370BFA, Puerto Iguazú, Misiones, Argentina.

^3^ Departamento de Estatística, Universidade Federal de São Carlos, CEP 13565-905, São Carlos, São Paulo, Brazil.

**Corresponding author:* Fagner Miguel da Silva; Address: Laboratório de Genética de Aves, Departamento de Genética e Evolução, Universidade Federal de São Carlos, Rodovia Washington Luís, km 235, CEP 13565-905, São Carlos, SP, Brazil; Tel: +55 16 3351 8391; E-mail: fagner.miguel.silva@gmail.com

**SUPPLEMENTARY TABLES**

**Table S1** Additional information for polymerase chain reactions (PCR) of microsatellites amplified in wood stork samples.

| PCR assay (volume)  Microsatellite locus | | Conc. of primers | Fluorescent dye (forward primer) | Annealing temperatures | Standard reaction and cycling profile |
| --- | --- | --- | --- | --- | --- |
| Multiplex I (15 µL) | |  |  | 55ºC (initial cycles), decreasing from 55ºC to 51ºC by touchdown approach (intermediate cycles), and 51ºC (final cycles). | Protocol of reactions: 54-60 ng of DNA template, forward and reverse primers (0.2-1.8 µM), 2.5 mM MgCl_2_, 0.42 mM dNTPs, 1x reaction buffer BD (Solis BioDyne) and 0.5 U HOT FIREPol® *Taq* DNA polymerase (Solis BioDyne).  Cycling profile: Preheating at 94ºC for 15 min, six initial cycles (94ºC for 30 s, initial annealing temperature for 45 s, and 72ºC for 30 s), twenty intermediate cycles (94ºC for 30 s, annealing temperature for 45 s with the touchdown approach, and 72ºC for 30 s), ten final cycles (94ºC for 30 s, final annealing temperature for 45 s, and 72ºC for 30 s), and a final polymerization at 72ºC for 10 min. |
|  | WSµ 18^1^ | 0.6 µM | 6-FAM |  |  |
|  | WSµ 20^1^ | 1.7 µM | TET |  |  |
|  | WSµ 23^1^ | 1.8 µM | 6-FAM |  |  |
|  | WSµ 24^1^ | 0.4 µM | 6-FAM |  |  |
| Multiplex II (12 µL) | |  |  | 58ºC (initial cycles), decreasing from 58ºC to 56ºC by touchdown approach (intermediate cycles), and 56ºC (final cycles). |  |
|  | WS4^2^ | 0.3 µM | 6-FAM |  |  |
|  | WSµ 09^1^ | 1.8 µM | 6-FAM |  |  |
| Multiplex III (12 µL) | |  |  | 58ºC (initial cycles), decreasing from 58ºC to 56ºC by touchdown approach (intermediate cycles), and 56ºC (final cycles). |  |
|  | WSµ 03^1^ | 1.1 µM | 6-FAM |  |  |
|  | WSµ 13^1^ | 0.2 µM | 6-FAM |  |  |
| Singleplex I (10 µL) | |  |  | 55ºC (initial cycles), decreasing from 55ºC to 51ºC by touchdown approach (intermediate cycles), and 51ºC (final cycles). |  |
|  | WS1^2^ | 1.0 µM | 6-FAM |  |  |
| Singleplex II (10 µL) | |  |  | 64ºC (initial cycles), decreasing from 64ºC to 62ºC by touchdown approach (intermediate cycles), and 62ºC (final cycles). |  |
|  | WS2^2^ | 1.0 µM | 6-FAM |  |  |
| Singleplex III (10 µL) | |  |  | 55ºC (initial cycles), decreasing from 55ºC to 49ºC by touchdown approach (intermediate cycles), and 49ºC (final cycles). |  |
|  | WS6^2^ | 1.0 µM | 6-FAM |  |  |
| Singleplex IV (10 µL) | |  |  | 55ºC (initial cycles), decreasing from 55ºC to 51ºC by touchdown approach (intermediate cycles), and 51ºC (final cycles). |  |
|  | WSµ 08^1^ | 1.0 µM | TET |  |  |
| Singleplex V (10 µL) | |  |  | 58ºC (initial cycles), decreasing from 58ºC to 56ºC by touchdown approach (intermediate cycles), and 56ºC (final cycles). |  |
|  | WSµ 14^1^ | 1.0 µM | HEX |  |  |

Species-specific microsatellite loci described by Tomasulo-Seccomandi et al. (2003)^1^ and van den Bussche et al. (1999)^2^.

**TABLE S2** Mean exclusive multilocus genotypes for simulated cohorts. Mean number of exclusive multilocus genotypes among individuals of the simulated cohorts (N = 800) from the dataset of the Porto da Fazenda (PF) wood stork population and subsets of different sizes (PF8, PF16, PF24, PF32 and PF40) across different numbers of loci.

| Genotype sets | Mean exclusive multilocus genotypes | | | |
| --- | --- | --- | --- | --- |
|  | 7 loci | 10 loci | 13 loci | 13 loci^1^ |
| PF8 | 459 | 792 | 800 | 800 |
| PF16 | 519 | 788 | 800 | 800 |
| PF24 | 520 | 788 | 800 | 800 |
| PF32 | 574 | 794 | 800 | 800 |
| PF40 | 630 | 796 | 800 | 800 |
| PF | 625 | 795 | 800 | 800 |

^1^Without adjusting allele frequencies for the presence of null alleles.

**REFERENCES**

Tomasulo-Seccomandi, A. M., Schable, N. A., Bryan Jr., A. L., Brisbin Jr., I. L., Del Lama, S. N., & Glenn, T. C. (2003). Development of microsatellite DNA loci from the wood stork (Aves, Ciconiidae, *Mycteria americana*). *Molecular Ecology Notes,* 3, 563-566.

van den Bussche, R. A., Harmon, S. A., Baker, R. J., Bryan Jr., A. L., Rodgers Jr., J. A., Harris, M. J., & Brisbin Jr., I. L. (1999). Low levels of genetic variability in North American populations of the wood stork (*Mycteria americana*). *The Auk*, 116, 1083-1092.
