# Supplementary figures and images for: Considerations for monitoring population trends of colonial waterbirds using the effective number of breeders and census estimates
Source: Ecol Evol. 2018 Jul 20;8(16):8088–101. doi: 10.1002/ece3.4347 (PMC6144984; doi:10.1002/ece3.4347)

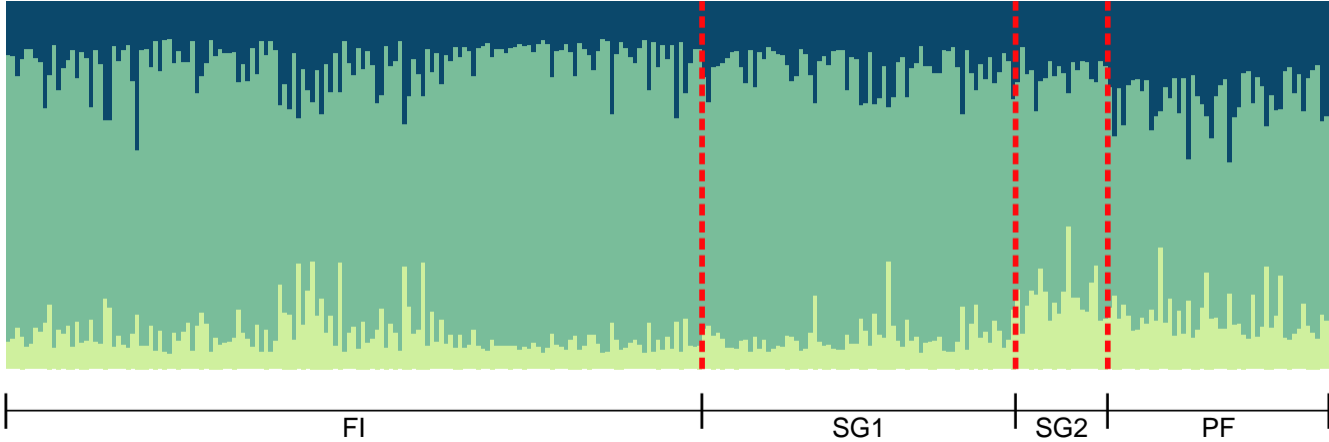

Supplement: Supplementary file 2 [file ECE3-8-8088-s002.pdf]

■ 7 loci    ● 10 loci    ▲ 13 loci

(A) ABC

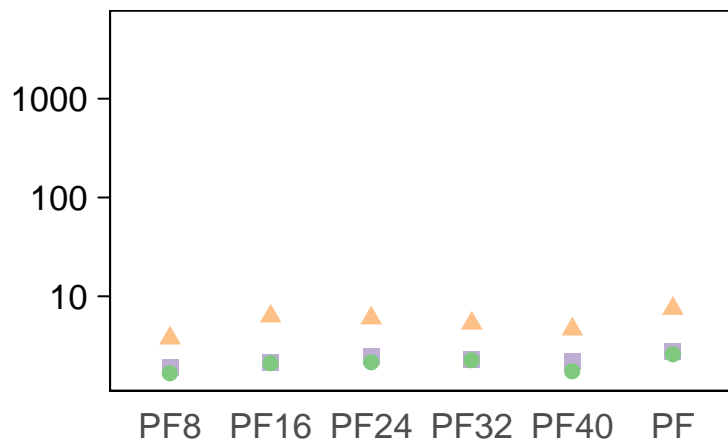

(B) MC

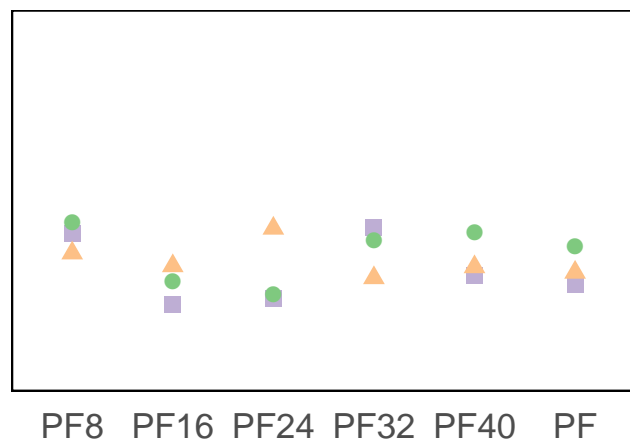

(C) SA

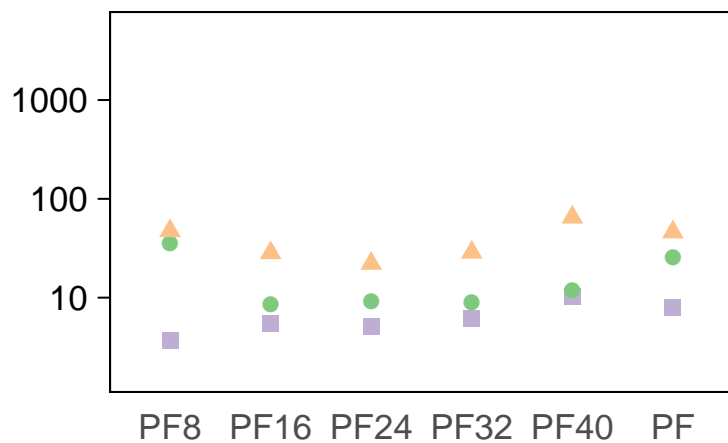

(D) HE bootstrap

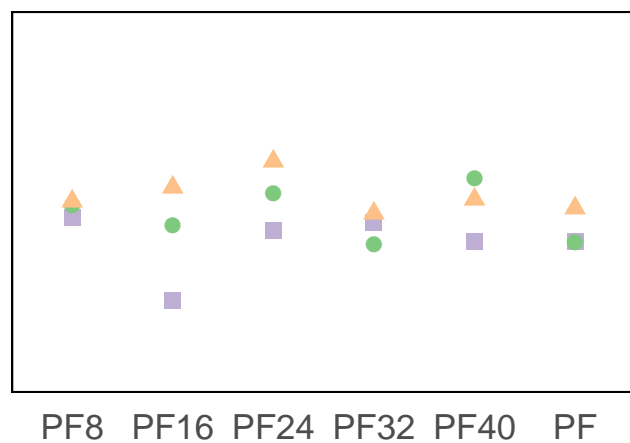

(E) LD > 0.05

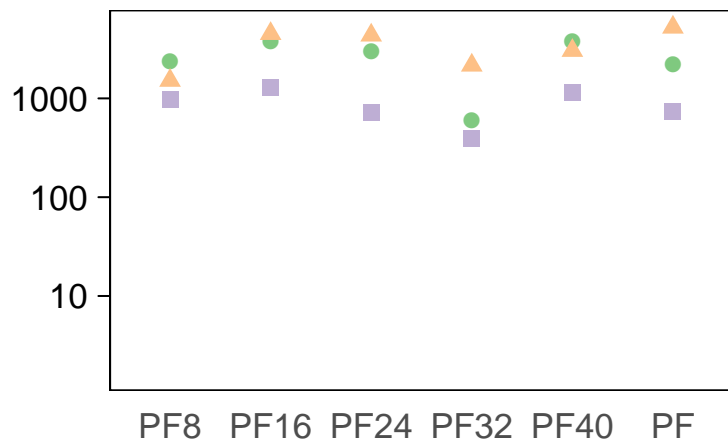

Supplement: Supplementary file 3 [file ECE3-8-8088-s003.pdf]
