# Supplementary material for: Considerations for monitoring population trends of colonial waterbirds using the effective number of breeders and census estimates
Source: Ecol Evol. 2018 Jul 20;8(16):8088–101. doi: 10.1002/ece3.4347 (PMC6144984; doi:10.1002/ece3.4347)
Supplement: Supplementary file 4 [file ECE3-8-8088-s004.docx]

**APPENDICES**

**Considerations for monitoring population trends of colonial waterbirds using the effective number of breeders and census estimates**

Fagner M. da Silva^1^*, Carolina I. Miño^2^, Rafael Izbicki^3^, Silvia N. Del Lama^1^

^1^ Departamento de Genética e Evolução, Universidade Federal de São Carlos, CEP 13565-905, São Carlos, São Paulo, Brazil.

^2^ Instituto de Biología Subtropical (IBS), Universidad Nacional de Misiones, CONICET, N3370BFA, Puerto Iguazú, Misiones, Argentina.

^3^ Departamento de Estatística, Universidade Federal de São Carlos, CEP 13565-905, São Carlos, São Paulo, Brazil.

**Corresponding author:* Fagner Miguel da Silva; Address: Laboratório de Genética de Aves, Departamento de Genética e Evolução, Universidade Federal de São Carlos, Rodovia Washington Luís, km 235, CEP 13565-905, São Carlos, SP, Brazil; Tel: +55 16 3351 8391; E-mail: fagner.miguel.silva@gmail.com

**APPENDIX 1** STRUCTURE clusters of wood stork populations from the Pantanal wetland. Individual membership proportions (vertical bars) to each of the clusters (*K* = 3), represented by colors. Individuals were grouped according to colony: Fazenda Ipiranga (FI), Sangradouro 1 (SG1), Sangradouro 2 (SG2) and Porto da Fazenda (PF). Simulations were performed with sampling location as prior information, admixture model, correlated allele frequencies, degree of admixture (α) and gamma distribution inferred from the data, 2x10^6^ run lengths, burn-in of 10^4^ and 20 iterations per *K* value.

**APPENDIX 2** Log root mean squared error (log RMSE) of estimates of effective number of breeders ($\hat{N}_{b}$) for simulated cohorts. Log RMSE was computed for $\hat{N}_{b}$ obtained for cohorts simulated from Porto da Fazenda (PF) population and subsets of different numbers of individuals genotyped (PF8, PF16, PF24, PF32 and PF40) with different sets of microsatellite loci using following methods: approximate Bayesian computation (ABC; A), molecular coancestry (MC; B), sibship assignment with sibship size prior and sibship scaling (SA; C), heterozygote excess with 10000 bootstrapping iterations (HE bootstrap; D), and unbiased linkage disequilibrium with allelic frequencies > 0.05 (LD > 0.05; E).

**APPENDIX 3** Confidence intervals (CIs) of 95% for estimates of effective number of breeders ($\hat{N}_{b}$) for simulated cohorts. $\hat{N}_{b}$ were obtained by different methods for cohorts simulated from Porto da Fazenda (PF) population and subsets of different numbers of individuals genotyped (PF8, PF16, PF24, PF32 and PF40) across different numbers of loci.

| Dataset | No. loci | Range of 95% CIs of $\hat{N}_{b}$^1^ | | | | |
| --- | --- | --- | --- | --- | --- | --- |
|  |  | ABC² | SA³ | MC^4^ | HE^5^ | LD^6^ |
| PF8 | 7 | 11.78-31.94 | 173-271 | 0-640.9 | 11.1-∞ | 151.8-∞ |
| PF8 | 10 | 13.08-35.11 | 201-413 | 0-925.5 | 13.8-∞ | 318.1-∞ |
| PF8 | 13 | 11.94-48.97 | 242-502 | 0.1-519.8 | 15.5-∞ | 542.8-∞ |
| PF16 | 7 | 12.45-41.13 | 170-271 | 0-150.5 | 14.5-∞ | 139.9-∞ |
| PF16 | 10 | 13.47-34.27 | 189-313 | 0-242.7 | 17.0-∞ | 254.0-∞ |
| PF16 | 13 | 15.67-69.41 | 227-433 | 0.1-361.6 | 16.1-∞ | 662.2-∞ |
| PF24 | 7 | 12.89-39.05 | 171-274 | 0-167.6 | 12.8-∞ | 133.2-∞ |
| PF24 | 10 | 13.61-34.99 | 191-315 | 0-235.9 | 20.1-∞ | 323.9-∞ |
| PF24 | 13 | 13.48-61.95 | 235-415 | 0-792.4 | 20.3-∞ | 773.7-∞ |
| PF32 | 7 | 12.12-36.41 | 169-274 | 0-773.5 | 12.9-∞ | 136.4-∞ |
| PF32 | 10 | 14.75-37.78 | 199-330 | 0-647.2 | 17.4-∞ | 353.9-∞ |
| PF32 | 13 | 14.19-59.62 | 222-431 | 0-252.8 | 20.9-∞ | 639.5-∞ |
| PF40 | 7 | 13.26-39.03 | 151-271 | 0-200.9 | 14.4-∞ | 181.1-∞ |
| PF40 | 10 | 15.89-3771 | 183-322 | 0-866.1 | 17.6-∞ | 339.4-∞ |
| PF40 | 13 | 17.46-59.31 | 171-539 | 0.1-412.9 | 21.3-∞ | 456.8-∞ |
| PF | 7 | 13.57-44.14 | 147-258 | 0-225.8 | 12.8-∞ | 139.6-∞ |
| PF | 10 | 16.30-40.04 | 133-299 | 0-534.4 | 14.3-∞ | 368.3-∞ |
| PF | 13 | 19.88-70.78 | 170-422 | 0-4191.6 | 18.9-∞ | 757.3-∞ |

^1^Finite $\hat{N}_{b}$ excluding outliers identified by boxplot method. ^2^Approximate Bayesian computation method (ONESAMP v1.2). ^3^Sibship assignment method (COLONY v2.0.6.1). ^4^Molecular coancestry method (NEESTIMATOR v2.0.1). ^5^Heterozygote excess method with 10000 bootstrap iterations (NB_HETEX v1.0). ^6^Unbiased linkage disequilibrium method with allelic frequencies > 0.05 (LDNE v1.31). ∞ = infinite value.
